# StUBC13, a Ubiquitin-Conjugating Enzyme, Positively Regulates Salt and Osmotic Stresses in Potato

**DOI:** 10.3390/ijms252313197

**Published:** 2024-12-08

**Authors:** Xue Fu, Xun Tang, Ning Zhang, Huaijun Si

**Affiliations:** 1 State Key Laboratory of Aridland Crop Science, Gansu Agricultural University, Lanzhou 730070, China; fuxue@st.gsau.edu.cn (X.F.); tangxun@gsau.edu.cn (X.T.); ningzh@gsau.edu.cn (N.Z.); 2 College of Agronomy, Gansu Agricultural University, Lanzhou 730070, China; 3 College of Life Science and Technology, Gansu Agricultural University, Lanzhou 730070, China

**Keywords:** ubiquitination, ubiquitin-conjugating enzyme, *StUBC13*, potato

## Abstract

Protein ubiquitination is an important regulatory mechanism for biological growth and development against environmental influences, and can affect several biological processes, including the growth, development, and stress responses of plants. However, the function of potato-related ubiquitin-conjugating enzymes in abiotic stress tolerance is poorly understood. In this study, a *StUBC13* with a UBC conserved structural domain was identified in potato and its function was investigated under osmotic stress and salt stress conditions. The observation of plant phenotypes under stress conditions revealed that overexpressed plants grew better than wild-type plants. In line with the above results, the determination of stress-related physiological indices revealed that the overexpression transgenic plants had better stress tolerance and stronger adaptation to environmental stress, and the transgenic plants were found to tolerate better drought and salt stress by decreasing their malondialdehyde (MDA) content and increasing their superoxide dismutase (SOD), peroxidase (POD), and catalase (CAT) contents under stress conditions. Based on these results, StUBC13 has an important regulatory role in the response of plants to abiotic stresses (osmotic stress and salt stress), and overexpression of this gene can improve the tolerance of potatoes to osmotic and salt stresses.

## 1. Introduction

The ubiquitin–proteasome system is an important pathway for protein degradation in eukaryotic cells and is involved in the degradation of 80–85% of eukaryotic proteins. Its function depends on three different enzymes: ubiquitin-activating enzyme E1, ubiquitin-conjugating enzyme E2, and ubiquitin-ligase enzyme E3. Ubiquitin-activating enzyme E1 activates ubiquitin molecules through ATP, then the activated ubiquitin molecules are delivered to ubiquitin-conjugating enzyme E2, and ubiquitin-ligase enzyme E3 recognizes the specific target proteins and transfers ubiquitin molecules to the substrates, which then degrade the substrate protein through the 26S proteasome to realize the degradation of the substrate protein [1]. Numerous studies have revealed that ubiquitination plays a regulatory role in the growth, development, and adversity stress of plants by maintaining the relative stability of intracellular proteins.

Depending on the ubiquitinated substrate, ubiquitin-conjugating enzyme E2 can directly bind to the substrate protein for protein degradation in order to exert degradation. Ubiquitin-conjugating enzyme E2 was first identified in rabbit reticulocyte schizonts and contains a UBC conserved structural domain that is highly consistent in composition across species, containing four α-helices, one β-fold, and one short peptide chain [2,3,4]. In recent years, it has been shown that UBC family genes play an important role in plants. The number of UBC family genes in different species has also been characterized, with there being 45 in Arabidopsis (*AtUBC1*-*AtUBC45*) [5], 59 in tomatoes (*SlUBC1*-*SlUBC59*) [6], 75 in maize (*ZmUBC1*-*ZmUBC75*) [7], 74 in bananas (*MaUBC1*-*MaUBC74*) [8], and 71 in soybeans (*GmUBC1*-*GmUBC71*) [9]. *AtUBCs* have been reported in Arabidopsis, where *AtUBC1/AtUBC2* and E3-type HUB1/HUB2, together, mediate H2B-type ubiquitination, which is involved in the activation of Arabidopsis floral repressor genes and the phenotypic diversification of mutants, among other processes [10]. AtUBC32 targeted to the endoplasmic reticulum membrane is involved in endoplasmic reticulum-associated protein degradation through the oleuropein lactone-mediated signaling pathway, which regulates plants’ tolerance to salt stress and growth regulation [11]. A ubiquitin-conjugating enzyme variant protein, COP10, inhibits seedling photomorphogenesis in the dark. In vivo, COP10 was able to form a CDD complex with DET1 and DDB1a, in parallel with its ability to enhance the ubiquitin-conjugating enzyme activity in vitro [12]. AtUBC13A and AtUBC13B interact with yeast or human Mms2 proteins and restore the sensitivity of yeast ubc13 deletion mutants to their mutations and DNA damage, playing an important role in DNA damage repair processes [13]. AtUBC2 localized in the nucleus and cytoplasm of *Arabidopsis thaliana* negatively regulates the plant’s disease resistance by modulating its SGT1 gene levels [14]. The functions of tobacco *NtUBC*-related genes have also been reported successively, and the heterologous expression of tobacco *NtUBC1* and *NtUBQ* in Arabidopsis thaliana was able to improve the salt tolerance of plants by enhancing the 26S proteasome activity and decreasing the Na^+^ accumulation and ROS accumulation [15]. Tobacco *NtUBC1* confers cadmium tolerance in tobacco plants through Ub/26S proteasome-dependent protein degradation or stimulating Ub-independent 20S proteasome degradation-associated protein [16]. Multiple *NbUBCs* in tobacco were found to be critical in the growth and development, immune response, and programmed cell death of plants [17]. *SlUBC3* can complement a yeast mutant lacking ubiquitin-conjugating enzymes and interacts with βC1 (a single-stranded satellite DNA). *SlUBC3* can reduce βC1 protein levels to fight plant diseases through ubiquitination s [18]. The heterologous overexpression of soybean *GmUBC2* in Arabidopsis thaliana revealed that it is involved in ionic homeostasis and the regulation of osmotic substance synthesis as well as oxidative stress responses that enhance the tolerance of the plant to drought and salt by responding to the expression of the proline biosynthesizing enzymes *AtNHX1* and *AtCLCa*, and the superoxide dismutase gene *AtCCS* [19]. *AhUBC2* was able to respond to abiotic stresses such as polyethylene glycol (PEG6000), salt, and low temperatures, and transformation of the gene into Arabidopsis revealed that the transgenic plants showed increased tolerance to water stress caused by sorbitol and natural drought. This may have resulted from activation of the ABA-independent signaling pathway, which specifically regulates stress-responsive genes and promotes the synthesis of osmoregulators to protect plants from water stress [20]. *GhUBC1* and *GhUBC2* identified from tetraploid cotton both showed a significant increase in expression during leaf and flower senescence, and the degradation of functional proteins during senescence retardation [21]. *VrUBC1* expression is induced by exposure to high amounts of salt, dehydration, and the exogenous abscisic acid (ABA); VrUBC1 interacts with a C3HC4-type RING E3 ligase, AtVBP1, which plays a regulatory role in osmotic stress by modulating ABA-related genes [22]. The four Chinese lotus *NnUBCs* homologous to *Arabidopsis thaliana* AtUBC1 and AtUBC2 possess different expression patterns in different tissues, and the transfer of NnUBC3 into the *Arabidopsis thaliana* mutant plants (*Atubc1*-*1Atubc2*-1) was able to restore them to the wild-type phenotype, suggesting that *NnUBC3* possesses the same gene function as *AtUBC1* and *AtUBC2* [23].

The potato is an annual herbaceous plant. With the growth of the global population, the potato plays an important role in guaranteeing food security and increasing the growth of national economies [24]. As an essential substance in plant growth and development, water is an important solvent in the transport of plant nutrients as well as in their absorption and utilization. Soil salinization is one of the major limiting factors affecting global agricultural development, and plants are severely endangered by the toxic and dehydrating effects of salt stress during their growth and development. Both drought and salt stress are common adversity stresses faced by plants. However, little is known about the regulatory role of the ubiquitin–proteasome system in potato resistance to abiotic stresses, especially regarding the function of the UBC gene.

Previously, we identified 57 *StUBC* genes in potatoes and found that *StUBC13* was involved in drought stress and salt stress in potatoes. In the present study, we cloned *StUBC13* from ‘Atlantic’ potatoes, investigated its expression pattern and subcellular localization, and verified its function in the stress response of plants by inciting its overexpression in potato. The observed increase in drought tolerance and salt tolerance may be due to the accumulation of the ubiquitin-conjugating enzyme StUBC13, which disrupts the normal expression of stress genes and ubiquitinates some stress-related proteins.

## 2. Result

### 2.1. Isolation and Characterization of StUBC13

**Figure 1:**
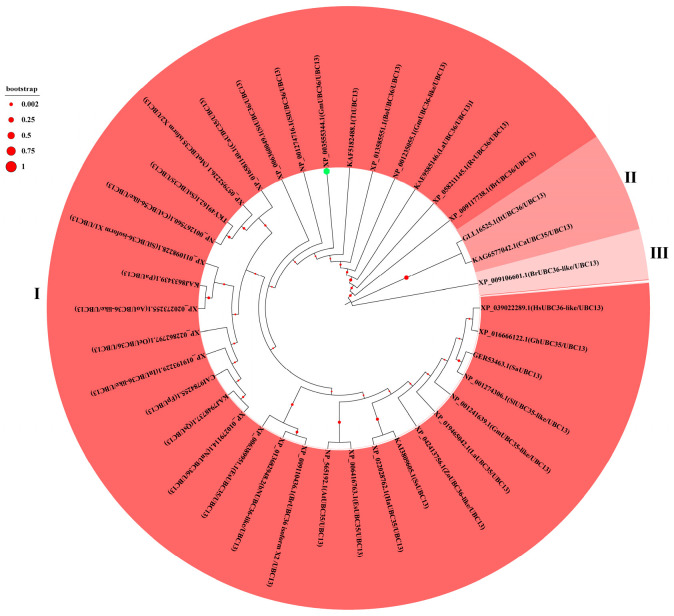
Phylogenetic analysis of homologs to StUBC13 in different species. The phylogenetic tree was constructed by MEGA 7.0 software using ClustalW for alignment and the neighbor-joining method for the construction of the phylogeny. One thousand replicates were used in bootstrap strap analysis to determine the support value for each branch. The green circle marks StUBC13. The accession numbers corresponding to the species names are as follows: XP_006360049.1 (*Solanum tuberosum*); NP_001274716.1 and NP_001274306.1 (*Solanum lycopersicum*); XP_009110436.1, XP_009106601.1, and XP_009117738.1 (*Brassica rapa*); XP_057952226.1 (*Malania oleifera*); GLL16525.1 (*Ipomoea trifida*); KAE9585146.1 (*Lupinus albus*); XP_010279114.1 (*Nelumbo nucifera*); XP_011098228.1 (*Sesamum indicum*); KAI3809605.1 (*Smallanthus sonchifolius*); NP_001241639.1, NP_001235055.1, and XP_003555344.1 (*Glycine max*); XP_006416763.1 and XP_006389951.1 (*Eutrema salsugineum*); KAF5182488.1 (*Thalictrum thalictroides*); XP_013585551.1 (*Brassica oleracea* var. *oleracea*); XP_019465042.1 (*Lupinus angustifolius*); XP_022862797.1 (*Olea europaea* var. *sylvestris*); XP_013682948.2 (*Brassica napus*); KAG6577042.1 (*Cucurbita argyrosperma* subsp. *sororia*); NP_001267560.1 (*Cucumis sativus*); KAJ7948737.1 (*Quillaja saponaria*); XP_042413756.1 (*Zingiber officinale*); XP_020273255.1 (*Asparagus officinalis*); XP_016581140.1 (*Capsicum annuum*); GER53463.1 (*Striga asiatica*); XP_022028762.1 (*Helianthus annuus*); XP_058211145.1 (*Rhododendron vialii*); XP_039022289.1 (*Hibiscus syriacus*); TKY49162.1 (*Spatholobus suberectus*); KAJ8633439.1 (*Persea americana*); XP_019193229.1 *(Ipomoea nil*); NP_565192.1 (*Arabidopsis thaliana*); XP_016666122.1 (*Gossypium hirsutum*); CAI9784255.1 (*Fraxinus pennsylvanica*).

A total of 57 *StUBC* genes were identified in potato in our previous study. In order to further investigate the function of the *StUBC13* gene, 38 UBC proteins homologous to it were obtained by NCBI’s Blast comparison and analyzed phylogenetically. The phylogenetic tree categorized the 38 proteins that are homologous to StUBC13 into three groups, in which group I contained 35 proteins, group II contained 2 proteins, and group III contained only 1 protein. Potato StUBC13 was categorized into group I, and in terms of its evolutionary relationship, StUBC13 is the closest relative to tomato (Figure 1).

### 2.2. Expression Analysis of StUBC13 in Potato

**Figure 2:**
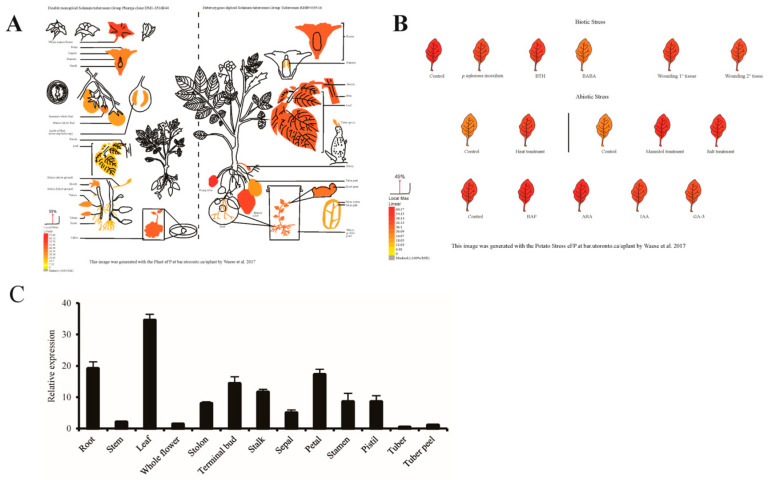
Relative expression levels of *StUBC13* in different tissues of potato. (**A**) Tissue-specific expression profiling of the *StUBC13* in DM and RH. (**B**) Expression profiling of *StUBC13* under different stress treatments. (**C**) Expression levels of *StUBC13* in different tissues were determined by qRT-PCR. The transcript values of the *StUBC13* in tuber peel were set to 1. Values are means ± SE of three replicates.

In order to further investigate the role of *StUBC13* in the growth and development of potato, we analyzed the expression of *StUBC13* in different tissues and organs and under stress conditions using previous reports on transcriptional data (http://www.bar.utoronto.ca/, accessed on 20 April 2024) from the potato database and analyzed the expression pattern of *StUBC13* in different tissues of the ‘Atlantic’ variety using quantitative real-time PCR (qRT-PCR). The expression pattern of *StUBC13* in different tissues of ‘Atlantic’ potato was analyzed by qRT-PCR. The transcript data showed that *StUBC13* was expressed at the highest level in whole flowers and induced by various biotic and abiotic stresses in ‘DM1-3516R44’ and ‘RH89-039-16’ (Figure 2A,B). The qRT-PCR results showed that *StUBC13* was expressed in different tissues and organs including the roots, stems, leaves, and flowers of ‘Atlantic’ potato, the highest expression was found in the leaves, which was 34.5 times higher than that in the tuber skin. The expression in the roots was 19 times higher than that in the tuber peels (Figure 2C). The above results suggest that *StUBC13* may play a role in nutrient uptake and energy conversion and that the expression of *StUBC13* can be induced under drought and salt stress-response conditions.

### 2.3. StUBC13 Gene Structure and Protein Sequence Analysis

**Figure 3:**
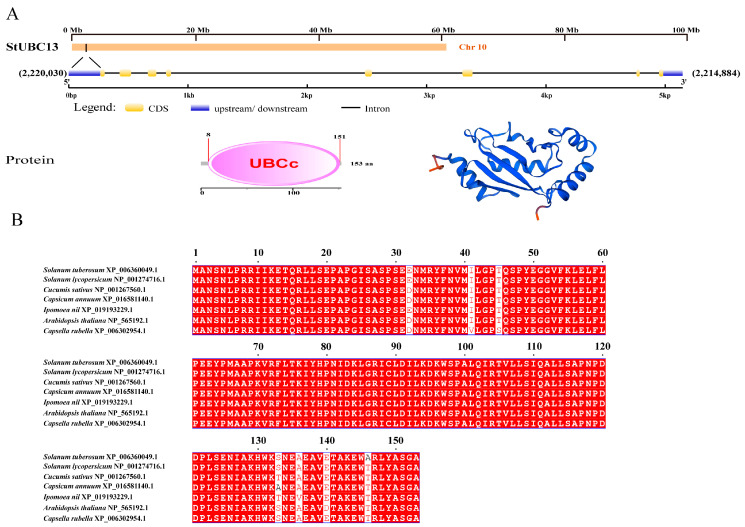
Protein sequence and gene structure analyses of StUBC13. (**A**) Intron-exon and conserved domain structures of StUBC13. (**B**) Amino acid sequence alignment of UBC13 in different species.

To determine the genetic information of *StUBC13*, we performed the gene structure analysis of *StUBC13* using genomic information. The results showed that *StUBC13* is located on chromosome 10, with a total length of 5147 bp, of which the CDs sequence is 462 bp long, encodes 153 amino acids, and contains eight exons and seven introns. Protein structure analysis revealed that StUBC13 contains an α-helix with irregular coiling (Figure 3A).

To further understand the evolutionary relationship between StUBC13 and UBC13Ss in other species, multiple sequence comparison analysis was performed, and it was found that StUBC13 is highly homologous to UBC13 proteins in other species and that all of them contain a UBC conserved structural domain, which is also conserved in other species (Figure 3B).

### 2.4. Overexpression of StUBC13 Is Regulating Osmotic Stress in Potato

**Figure 4:**
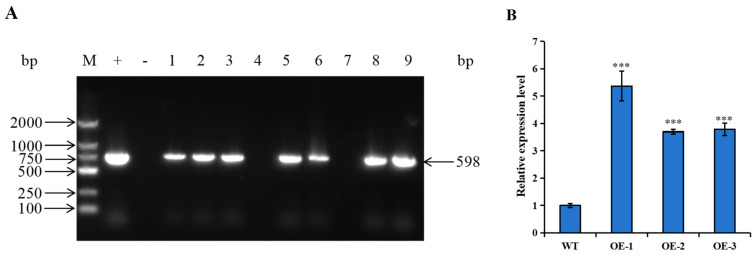
Identification of the *StUBC13* transgenic plants. (**A**) Detection at the DNA level of transgenic lines using PCR. (**B**) Relative expression of *StUBC13* in leaves of both WT and *StUBC13*-OE lines. The WT expression data in leaves were normalized to 1. (***) *p* < 0.001.

To elucidate the biological function of *StUBC13*, we introduced the CDS sequence of *StUBC13* into Atlantic potato plants by constructing an overexpression vector to obtain overexpression transgenic lines. The positive plants that grew normally in a resistance medium containing hygromycin B were examined at the DNA level, and a total of seven transgenic lines were obtained (Figure 4A). We randomly selected three transgenic lines for further study. The expression levels of *StUBC13* in the leaf tissues of wild-type (WT) and three overexpression plants (OE-1, OE-2, and OE-3) were detected by qRT-PCR. The results showed that *StUBC13* was significantly expressed in the overexpression lines compared with the WT (Figure 4B). In the overexpression plants, OE-1, OE-2, and OE-3, the relative expressions of *StUBC13* were 5.37, 3.70, and 3.79 times higher than that of the WT plants, respectively.

**Figure 5:**
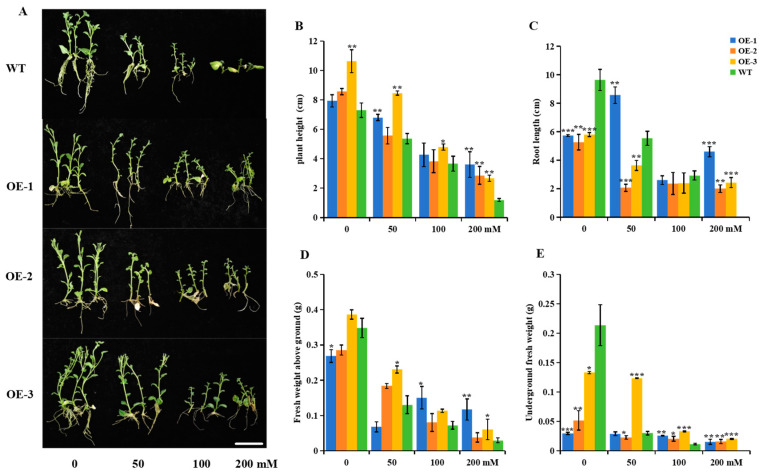
Overexpression of *StUBC13* improves osmotic stress tolerance in potato. (**A**) Phenotypic characterization of osmotic stress tolerance in potato overexpressing *StUBC13* for 45 days under laboratory conditions. (**B**) Comparison of plant height after 45 days of mannitol incubation. (**C**) Root length comparison after 45 days of mannitol culture. (**D**,**E**) Biomass of WT and OEs under different mannitol stresses. Scale bars, 5 cm. All determinations were carried out on three biological replicates, with 10 plants per condition. Data are expressed as the mean ± SD; (*) *p* < 0.05; (**) *p* < 0.01; (***) *p* < 0.001.

Mannitol, a common osmoregulator, does not cause hypoxia in plant roots during stress. It is non−toxic and has a low molecular weight. Due to these properties, mannitol can provide plants with different drought stress conditions. In order to analyze the role of *StUBC13* in drought stress conditions, the plants were subjected to drought−stimulated stress using mannitol. The WT and OE-1, OE-2, and OE-3 were propagated in MS medium containing different concentrations of mannitol (0, 50, 100, and 200 mM), and their agronomic traits and stress-related indices were statistically analyzed under the different mannitol stress conditions. The results showed that the overexpression lines significantly increased the plant height under the no-stress condition (0 mM) compared with the WT, but the root length and biomass of the underground parts were significantly lower than those of the WT. With the increase in the mannitol concentration, the plant height, root length, and biomass accumulation of the WT and OE plants decreased. Especially under high mannitol stress (200 mM), the WT plants showed no signs of rooting and almost stopped growing. However, the OE plants showed good growth (Figure 5A–E).

**Figure 6:**
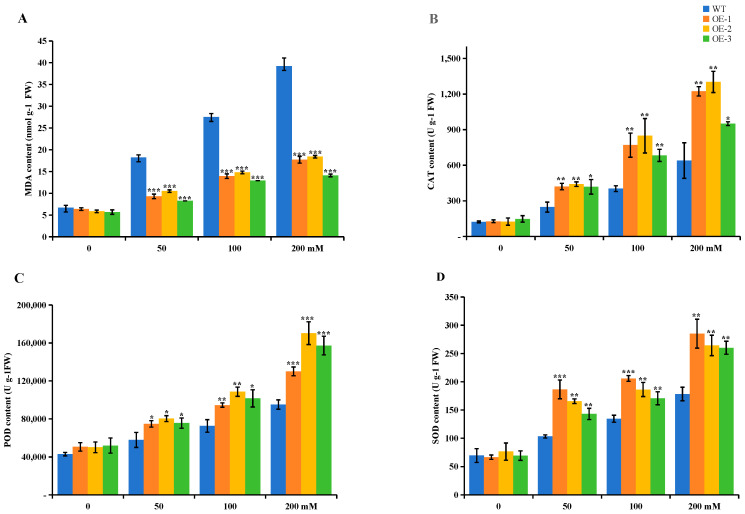
Effects of osmotic stress on MDA content and SOD, POD, and CAT activities in potato leaves. (**A**) Analysis of MDA content. (**B**) Analysis of CAT activity. (**C**) Analysis of POD activity. (**D**) Analysis of SOD activity. The left is the water treatment group; the right is the mannitol treatment group. Each datum value is from three replicated experiments. The SD is represented by an error bar (*n* = 3). Significant differences were denoted by *, meaning *p* < 0.05; **, meaning *p* < 0.01; ***, meaning *p* < 0.001.

SOD, POD, and CAT are important substances for plant cells to maintain their balance of reactive oxygen species metabolism, and they are mainly responsible for scavenging reactive oxygen species components from the body and protecting the plant from damage. To further verify the reliability of the results, we determined the changes in these three important antioxidant enzymes, SOD, POD, and CAT, as well as MDA content. SOD, POD, and CAT complement each other in plant adversity stress conditions and maintain cellular homeostasis by scavenging intracellular free radicals to ensure the normal growth of the plant [25]. The results are summarized in the following table. Under stress-free conditions (0 mM), there were no significant changes in the physiological indices of individual potato plants, but the changes in physiological indices varied greatly under osmotic stress and were significantly different among the collected data. The osmotic stress resulted in an increase in the activities of three antioxidant enzymes (SOD, POD, CAT) and in the MDA content in the plants. Relative to the wild-type plants, the MDA content was significantly lower in the overexpression plants (Figure 6A), in contrast to the SOD, POD, and CAT activities, which were significantly higher in the wild-type plants (Figure 6B–D). The physiological indexes measured under osmotic stress simulated by different concentrations of mannitol indicated that the overexpression of *StUBC13* could enhance the tolerance of potato plants to osmotic stress.

### 2.5. Overexpression of StUBC13 Regulates Salt Stress in Potato

**Figure 7:**
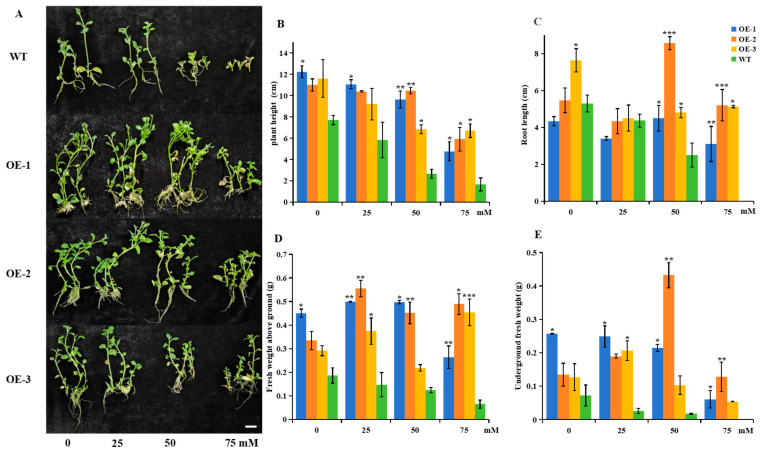
Statistics for the agronomic traits of OEs and WT. (**A**) Comparison of phenotypes among OE and WT plants. (**B**,**C**) Plant height and root length of WT and OEs under different salt stresses. (**D**,**E**) Biomass of WT and OEs under different salt stresses. Scale bars, 2 cm. All determinations were carried out on three biological replicates, with 10 plants per condition. Data are expressed as the mean ± SD; (*) *p* < 0.05; (**) *p* < 0.01; (***) *p* < 0.001.

To further determine its biological functions during salt stress, the role of *StUBC13* in salt stress was evaluated by comparing the phenotypic data and physiological and biochemical indices of the WT and OE plants under different concentrations of NaCl (25, 50, 75 mM) treatments. Under normal growth conditions (0 mM), the OE plants showed higher root lengths, plant heights, aboveground biomass, and belowground biomass accumulation than the WT plants (Figure 7). 

Similar to the results of the osmotic stress experiment, the physiological indices (root length, plant height, aboveground biomass, and belowground biomass) of the WT and OE plants decreased along with the increasing NaCl concentration. The WT plants showed a 65.32% decrease in plant height, a 52.83% decrease in root length, and a 34.87% decrease in aboveground fresh weight after the salt treatment (50 mM) as compared to the control sample (0 mM). However, the plant heights of the OE plants decreased by 21.25%, 5.45%, and 41.12% after the salt treatment (50 mM) as compared to the control sample (0 mM). The root length rose in the range of 4.65–57.4%. The aboveground biomass accumulation increased in the range of 11.1–34.7%. Under the higher concentration of NaCl (75 mM) stress, the root systems of the WT plants did not show any signs of growth, and, for all WT plants, the whole plant almost stopped growing, whereas the OE plants showed good growth phenomena both in terms of their root growth and the growth of the aboveground parts of the plants.

Under the no salt stress condition (0 mM), there were no significant changes in the physiological indices of the WT and OE plants, but the physiological indices of the potato strains under the salt stress condition were changed, and the variability between the data was significant. The salt stress resulted in increased MDA content and increased SOD, POD, and CAT activities in all lines. The SOD, POD, and CAT activities of the OE plants were significantly higher than those of the WT plants under the salt stress conditions; however, the MDA contents of the OE plants were significantly lower than those of the WT plants. The above results indicate that the overexpression of *StUBC13* could elicit changes in reactive oxygen species in vivo to improve the salt tolerance of plants.

## 3. Discussion

When plants are subjected to adverse stress conditions, a large number of misfolded proteins or substances that are toxic to the organism will be produced in the cell body, and the accumulation of these substances will damage the integrity of the cell structure and affect the normal metabolic process of the cell, thus leading to the impediment of plant growth and development. However, in vivo, most of these misfolded proteins are specifically degraded by the ubiquitin–proteasome system, which maintains the normal metabolic processes of the organisms and ensures their survival. Therefore, the expression of some key enzymes in the ubiquitin-conjugating enzyme system is closely related to the stress tolerance of plants, and ubiquitin-conjugating enzymes have the function of binding and delivering ubiquitin molecules to target proteins throughout the entire ubiquitination process, which is important for the specificity and precision of the ubiquitination modification process.

Two ubiquitin-conjugating enzymes, E2 (UBC) and E2-like (UEV), are present in the plant genome, and these determine the efficiency and specificity of target protein ubiquitination. Ubiquitin-conjugating enzyme E2 contains a conserved catalytic structural domain consisting of 150 amino acids and a conserved cysteine site; UEV, on the other hand, only has a conserved UBC structural domain and no ubiquitin-conjugating enzyme activity. The protein encoded by the StUBC13 cloned in this study has a high degree of similarity to and consistency with the protein sequences of other species, all of which have a cysteine catalytic site and ubiquitin-conjugating enzyme activity; thus, StUBC13 belongs to the category of ubiquitin-conjugating enzyme genes [10].

The qRT-PCR results showed that *StUBC13* was expressed in several tissues of potatoes, including the roots, stems, leaves, and flowers, suggesting that it may be involved in a variety of physiological and biochemical processes in plants. Especially, its expression was higher in the roots and leaves, which may improve plants’ stress tolerance by regulating their root development and increasing their photosynthetic efficiency. Transcriptome analysis revealed that *StUBC13* was induced by ABA and IAA hormones, which implies that *StUBC13* is involved in the transduction of the ABA signaling pathway, which plays an important role in the growth and development, as well as the stress response, of plants. Studies have shown that plants can respond to abiotic stresses (drought, salt, and low temperature) by accumulating or decreasing their ABA content in response to stress [26].

Drought stress is the most important cause of crop yield loss and quality loss. It is estimated that the annual crop yield loss due to drought exceeds the total economic loss in agriculture due to other causes. Crops are not singularly affected by drought stress, but also experience a series of other stress responses, such as oxidative stress, osmotic stress, cellular metabolism disorders, etc. [27,28]. The antioxidant stress system avoids the massive accumulation of ROS in plants under stress conditions by clearing free radicals. Therefore, it plays an important role in the tolerance of abiotic stresses [29]. Under osmotic stress and salt stress conditions, the activities of SOD, POD, and CAT were significantly increased in transgenic lines compared to the WT plants, indicating that *StUBC13* alters plant stress tolerance by regulating the activities of ROS-related enzymes under abiotic stress conditions.

**Figure 8:**
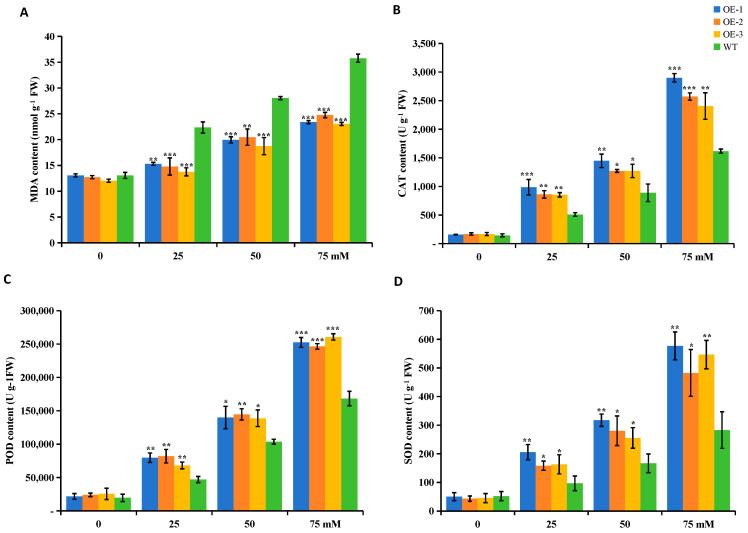
Analysis of MDA (**A**), CAT (**B**), POD (**C**), and SOD (**D**) in potato using WT indicators as controls. The SD is represented by an error bar (*n* = 3). (*) *p* < 0.05; (**) *p* < 0.01; (***) *p* < 0.001.

Salt stress is second only to drought stress in its impact on agricultural production. When plants are subjected to salt stress, the salt ions retained in the soil can directly interact with the plant root system, resulting in the formation of osmotic pressure between the root cells and the external environment, which affects the absorption and utilization of water, photosynthesis, and the metabolism of proteins and lipids in the organism [26]. In this study, the agronomic traits of potato OE and WT plants were analyzed under different concentrations of salt stress conditions. The results, as shown in Figure 8, showed that the OE plants exhibited good stress tolerance over WT plants in stress environments, while the overexpressed plants in 75 mM NaCl still showed good growth. It was found that the accumulation of Na^+^ at lower concentrations could improve the tolerance of plants to salt stress [30]. Ionic homeostasis in the cell is usually maintained in the plant body by regulating the relative balance of Na^+^ and K^+^ [31]. The heterologous expression of tobacco *NtUBC1* in *Arabidopsis thaliana* revealed that the overexpression plants possessed lower concentrations of Na^+^ and higher concentrations of K^+^, thereby improving the tolerance of transgenic Arabidopsis to salt stress [15].

Additionally, StUBC13 may play a more direct role by delivering ubiquitin molecules straight to target proteins within the nucleus or cytoplasm. This direct action leads to the ubiquitination of stress-responsive proteins, effectively regulating their abundance and activity.

Research on the plant ubiquitin-conjugating enzyme system has become a hot topic, especially the ubiquitin-conjugating enzymes, which plays a bridging role in ubiquitination, but research on the function of ubiquitin-conjugating enzymes is still at an initial stage in most plants except *Arabidopsis thaliana*. Existing reports show that ubiquitin-conjugating enzymes play an important role in plant stress and development, but little is known about how potato ubiquitin-conjugating enzymes respond to stress, so it is necessary to further investigate the function of these genes in potato ubiquitin-conjugating enzymes.

## 4. Materials and Methods

### 4.1. Plant Materials and Growth Conditions

Potatoes (*Solanum tuberosum* L. variety ‘Atlantic’) were grown in a greenhouse at 23 ± 2 °C with a photoperiod of 16 h/8 h (day/night). Tissue organs such as roots, stems, leaves, and flowers of WT potato plants at flowering stage (6 weeks after germination) were collected for tissue-specific expression analysis. The collected samples were immediately frozen in liquid nitrogen and stored at −80 °C for subsequent experiments.

### 4.2. Bioinformatics Analysis

Amino acid sequences of different species with more than 98% homology were obtained by comparison with NCBI and based on known StUBC13 sequence information. Phylogenetic evolutionary tree was constructed using MEGA 7.0 software. Multiple sequence comparison of StUBC13 in different species was conducted using DNAMAN V6 software. Online website Gene Structure Display Server (GSDS: http://gsds.cbi.pku.edu.cn, accessed on 22 April 2024) was used to perform StUBC13 gene structure analysis.

### 4.3. qRT-PCR Assay

RNA was extracted from different tissues and organs of potato roots, stems, leaves, and flowers at flowering stage, and cDNAs of different tissue samples were synthesized using Quant cDNA First Strand Synthesis Kit (TIANGEN, Beijing, China). cDNAs of different tissue samples were analyzed using SuperReal PreMix Plus (SYBR Green) kit (TIANGEN, Beijing, China) and the Light Cycler 480 system (Roche, Switzerland) for qRT-PCR analysis. *StEF1α* (GenBank ID: AB061263.1) was used as an internal reference gene. The primer sequences are shown in Supplementary Table S1.

### 4.4. Overexpression Vector Construction and Plant Transformation

The CDS removal terminator sequence of *StUBC13* (GenBank ID: XM_006359987.2) was amplified and ligated into the pCAMBIA1300-35S-EGFP vector, driven by the CaMV 35S promoter. The recombinant plasmid was transformed into GV3101, and transgenic plants were obtained by the potato block transformation method and the flower dip method, respectively [32,33]. The transformed plants were screened in MS (Murashige-Skoog) medium containing hygromycin B, and the transgenic plants were verified by PCR. The primer sequences are shown in Supplementary Table S1.

### 4.5. Phenotypic Analysis and Determination of Physiological and Biochemical Indices of Overexpressed StUBC13 Plants

To verify the tolerance of *StUBC13* under osmotic stress and salt stress conditions, we used a histoculture stress experiment. OE and WT stem segments with leaves were propagated in MS medium containing different concentrations of salt and mannitol and maintained in the conditions required for potato growth and development (25 °C, 16/8 h) for one month. Photographs were taken after one month and leaf tissues were collected for analysis. At least three replicates of each group of material were obtained.

SOD, POD, and CAT activities and MDA content were determined by obtaining enzyme crude extracts of leaf tissues using the matching extracts according to the manufacturer’s instructions (YX-W-A500 for SOD, YX-W-A502 for POD, YX-W-A501 for CAT, YX-W-A401 for MDA). Specifically, POD reacted with hydrogen peroxide to oxidize guaiacol and the absorbance value at 470 nm was measured to calculate POD activity. Hydrogen peroxide has a characteristic absorbance value at 240 nm and CAT is able to decompose hydrogen peroxide; therefore, CAT activity was calculated based on the change in absorbance value. MDA condenses with thiobarbituric acid to form a red product; the absorbance values at 532 nm and 600 nm were measured and the difference was used to calculate the content of MDA.

### 4.6. Statistical Analysis

Experimental data were analyzed and corresponding graphs were plotted using Microsoft Excel 2010 (Microsoft Corp., Seattle, WA, USA). Three replications were performed for each set of experiments. Values are presented as mean ± standard deviation. Data were analyzed using one-way ANOVA, with a significance level set at *p* = 0.05.

## 5. Conclusions

*StUBC13* is a ubiquitin-conjugating enzyme isolated from ‘Atlantic’ potato that responds to a variety of stress conditions. The gene is expressed in all potato tissues, with the highest relative expression being in the leaves. By constructing potato transgenic plants that overexpress *StUBC13* and analyzing their phenotypes and physiological and biochemical indexes under stress conditions, it was found that the overexpression of *StUBC13* improved the survival ability of potato plants under salt stress and osmotic stress. This analysis of the function of *StUBC13* can provide the possibility of the molecular breeding of potato.

## Data Availability

All data are available within the manuscript.

## Supplementary Materials

The following supporting information can be downloaded at: https://www.mdpi.com/article/10.3390/ijms252313197/s1.
