# Supplementary material for: StUBC13, a Ubiquitin-Conjugating Enzyme, Positively Regulates Salt and Osmotic Stresses in Potato
Source: Int J Mol Sci. 2024 Dec 8;25(23):13197. doi: 10.3390/ijms252313197 (PMC11642293; doi:10.3390/ijms252313197)
Supplement: Supplementary file 1 [file ijms-25-13197-s001.zip › ijms-3292523-supplementary.pdf]

Supplementary Table S1 Primer information used in this study

	Primers (5'-3')
Vector construction for overexpression lines	
StUBC13-EGFP-F	GAGCTCGGTACCCGGGGATCCATGGCTAACAGCAATCTTCCTCGA
StUBC13-EGFP-R	GCCCTTGCTCACCATGTCGACTCATGCACCACTAGCATATAGGCG
Verification of transgenic plants	
Hyg-F	GCTTCTGCGGGCGATTTGTGT
Hyg-R	GGTCGCGGAGGCTATGGATGC
Vector construction for qRT-PCR analysis	
ef1α-F	CGACTGTCACACTTCCCACA
ef1α-R	CCTCACAGCAAAACGACCCA
qPCR-F	TGCTCTTCAGATTCGTACCGT
qPCR-R	GACGCGCCCATTCTTTAGC
